# Supplementary material for: Surface Microbiome Profiling of Dental Elevators Using Third‐Generation Sequencing: Implications for Infection Control in Dental Practice
Source: Microbiologyopen. 2025 Dec 8;14(6):e70178. doi: 10.1002/mbo3.70178 (PMC12685768; doi:10.1002/mbo3.70178)
Supplement: Supplementary file 1 — customer_backup. [file MBO3-14-e70178-s001.zip › customer_backup/customer_backup/taxa_summary/krona/groups/treat/M04.Krona.html]

Javascript must be enabled to view this page.

members
magnitude
magnitudeUnassigned

M04.krona

64805

64793

67

0

0

0

0

0

0

0

0

0

0

0

0

0

0

0

67

67

67

67

0

25

13

0

10

6

0

0

13

0

0

0

0

0

0

0

0

0

0

0

0

0

0

8

2

0

0

0

0

1

1

0

0

1

1

0

0

0

0

0

0

0

1

1

1

1

0

0

0

0

0

0

0

0

0

0

0

0

0

0

0

0

0

0

0

0

0

0

0

0

0

0

0

0

6

4

4

4

4

0

0

0

2

1

1

1

0

0

0

0

0

0

0

0

0

1

1

1

34

31

31

0

0

0

31

2

2

0

29

23

1

5

0

0

0

2

0

0

0

0

2

0

0

0

0

0

0

0

0

0

0

0

0

0

2

0

0

0

0

0

0

0

0

0

0

0

0

0

2

2

0

0

0

0

0

0

0

0

0

0

0

0

0

0

0

1

1

1

0

0

0

0

1

1

0

0

0

0

0

0

0

0

0

0

0

0

0

0

0

0

0

0

0

0

0

0

0

0

0

0

0

0

0

0

0

0

0

0

0

0

0

0

0

0

0

0

0

0

0

0

0

0

0

0

0

0

0

0

0

0

0

0

0

0

0

0

0

0

0

0

0

0

0

0

0

0

0

0

0

0

0

0

3

3

3

0

0

0

0

0

0

3

3

3

0

0

0

0

0

0

495

163

163

163

10

10

86

86

47

8

39

20

9

11

24

24

24

24

24

308

308

308

70

70

0

217

0

12

100

0

105

14

14

7

7

0

443

111

111

0

0

0

111

111

111

0

0

0

0

0

0

0

0

0

0

0

0

0

0

0

0

332

138

130

0

0

0

0

130

0

84

46

0

8

8

8

0

0

0

0

0

0

0

0

0

0

123

68

6

6

15

15

47

25

22

0

0

0

0

0

0

0

46

0

0

0

46

0

46

0

0

9

9

1

8

71

71

14

14

0

0

0

0

57

3

54

0

0

0

0

0

0

0

14745

9081

0

0

0

0

321

32

32

32

244

0

0

0

12

0

12

10

10

222

0

222

0

45

45

45

112

112

88

0

88

24

24

0

0

7

6

6

6

1

0

0

0

0

0

0

1

1

0

0

0

64

64

64

3

0

61

0

0

0

0

0

0

0

0

0

0

0

0

4896

4896

8

8

266

21

0

58

7

180

0

0

69

69

0

0

133

133

5

5

5

5

0

0

22

0

0

0

6

16

0

0

0

0

755

0

461

121

173

0

0

0

3549

0

0

0

82

0

1

0

0

0

2

0

6

297

71

7

0

7

905

766

0

1169

0

0

27

202

0

0

0

0

7

84

1

0

0

83

17

9

9

9

8

8

8

0

0

2047

186

0

0

15

15

0

11

11

0

0

0

27

27

0

133

29

0

104

9

9

9

133

17

0

17

64

0

14

6

0

0

44

0

0

52

52

0

0

0

0

0

0

1

1

1

0

0

0

7

7

7

495

3

3

36

36

0

0

0

1

1

0

134

134

0

12

12

0

0

0

0

0

0

0

248

44

39

123

0

0

4

38

6

6

55

6

23

0

14

0

0

0

0

0

10

1

1

0

0

88

0

0

1

1

53

53

0

0

34

34

3

3

3

1

1

1

0

0

0

0

0

0

83

11

3

0

8

0

0

0

9

9

63

0

18

34

11

40

22

21

1

18

18

0

230

10

3

7

35

35

94

94

1

1

0

0

0

0

0

24

24

0

0

66

21

45

0

0

0

0

771

312

312

8

8

2

2

18

18

0

0

0

0

364

32

15

13

0

139

28

31

56

9

0

18

23

0

0

27

27

14

3

2

0

9

0

0

0

0

0

0

26

26

0

0

0

0

0

0

213

0

0

0

0

0

213

95

8

5

50

32

33

7

26

27

0

1

26

0

13

13

45

0

45

0

7

7

7

7

26

26

22

0

0

22

4

0

4

0

0

0

0

0

0

0

0

0

0

0

0

0

0

0

30

30

30

30

0

0

0

0

0

0

0

0

0

0

0

0

0

0

0

0

0

0

0

98

98

0

0

37

0

37

13

13

18

14

0

0

0

4

0

0

0

0

0

1

1

0

0

0

0

29

22

0

7

0

0

0

0

6

6

6

6

209

10

10

10

0

0

0

0

0

0

22

22

22

150

150

150

0

0

0

0

0

0

4

4

4

23

23

23

1028

1028

0

0

9

9

0

0

0

0

51

51

0

9

9

0

0

0

0

0

0

0

0

0

0

1

1

13

13

0

0

0

0

0

33

4

11

18

0

0

867

20

313

534

0

0

0

0

45

0

21

24

5664

0

0

0

0

0

16

16

6

6

10

10

0

0

0

0

0

0

0

0

3289

0

0

0

0

0

29

0

0

0

0

29

29

9

0

0

0

9

9

0

0

0

0

0

0

7

7

7

0

0

0

49

49

49

0

0

1688

0

0

0

0

494

494

464

9

8

42

405

730

0

49

15

0

28

409

52

26

16

41

0

1

40

7

11

35

158

0

0

6

6

91

0

80

11

22

22

0

0

39

0

39

0

0

0

61

0

0

0

0

0

0

37

0

0

37

6

0

6

0

18

18

5

4

4

0

0

0

0

0

1

0

0

1

72

72

72

1208

0

0

31

0

2

29

25

25

13

13

84

84

118

98

0

20

540

87

453

0

0

59

0

59

0

0

32

0

32

73

39

34

0

1

1

11

0

11

221

221

0

0

3

0

0

3

0

3

34

30

18

17

1

12

12

0

0

4

4

0

4

0

0

0

0

0

0

0

0

0

0

0

0

0

0

0

0

0

0

0

0

0

0

0

0

0

0

0

0

0

0

0

0

0

0

0

0

0

0

0

64

13

13

13

18

2

1

1

0

0

0

0

0

0

16

0

1

6

9

0

0

0

0

0

0

0

0

0

0

0

0

0

0

0

0

0

0

0

0

0

25

25

1

0

19

5

8

0

0

0

0

0

0

0

0

0

0

0

0

0

8

8

0

0

0

0

0

0

0

0

0

0

0

0

0

0

0

0

0

0

0

0

0

0

0

0

0

0

12

0

0

0

12

0

0

0

0

12

0

12

6

6

6

6

334

1

1

1

0

0

0

0

0

3

0

0

0

3

3

0

0

0

78

11

11

5

5

6

6

56

56

22

22

20

2

213

213

213

0

0

0

1

1

1

16

0

0

0

0

1

1

0

0

0

0

0

0

0

15

15

0

0

0

0

0

0

0

0

0

0

0

0

5

5

5

0

0

0

0

0

0

0

5

0

0

0

0

0

0

0

0

0

0

0

1800

385

64

64

34

0

34

287

287

0

0

0

0

0

1415

3

3

4

0

4

0

0

0

0

38

0

21

5

12

1370

0

0

387

3

5

0

502

41

46

0

1

29

356

0

0

0

0

0

0

0

0

0

0

0

0

0

0

0

0

0

0

0

0

0

0

17

17

11

11

0

0

0

0

0

0

0

0

6

6

0

0

0

0

0

0

0

0

0

0

1

1

1

1

86

6

6

6

0

0

0

80

80

80

0

0

0

0

0

0

0

0

0

0

0

0

613

436

436

0

0

0

436

27

27

0

0

41

41

30

30

33

0

33

82

82

5

5

218

168

39

11

177

0

0

0

0

0

0

0

9

9

9

9

0

0

0

0

0

0

75

44

0

0

0

0

0

0

0

0

0

44

44

0

0

0

0

0

0

0

0

0

17

7

4

3

10

0

0

10

0

14

0

0

14

14

0

0

0

0

76

76

76

0

0

0

76

0

0

0

0

0

0

0

0

0

0

0

0

0

0

0

17

17

4

4

13

13

47

47

47

47

47

0

33

8

6

0

0

0

0

0

0

1418

0

0

0

0

0

1418

1034

9

9

9

1005

0

0

0

31

1

29

1

3

0

3

0

0

13

0

13

0

0

0

0

0

524

30

16

17

0

0

457

1

0

3

160

0

96

64

0

0

1

1

0

0

49

15

0

34

22

22

0

0

106

84

22

0

96

96

20

20

0

9

11

0

0

0

5

0

0

0

2

0

0

0

0

0

0

0

0

0

0

0

0

0

0

0

0

0

0

1

0

1

1

1

0

0

0

0

0

0

0

0

0

0

0

0

0

0

0

0

0

0

0

0

0

0

0

0

0

0

0

0

0

0

0

3

0

0

0

0

3

3

0

0

135

0

0

0

0

0

0

0

0

0

0

0

18

0

0

5

5

3

3

10

10

18

0

0

0

0

0

0

0

0

0

0

0

2

2

16

0

16

0

0

0

0

0

0

0

0

0

0

0

1

0

0

0

0

1

0

0

1

0

0

0

0

0

0

0

0

27

27

0

0

0

4

8

0

0

0

0

0

12

0

0

0

0

0

3

7

1

1

1

0

1

5

2

2

0

0

0

1

64

0

0

0

1

0

1

37

0

0

5

0

32

1

1

0

0

0

0

25

0

0

0

13

0

6

0

0

0

0

0

0

0

0

6

0

0

0

0

0

0

0

0

0

0

0

0

0

0

0

0

0

0

0

0

0

0

0

0

0

0

0

0

0

0

0

0

0

0

0

0

0

0

0

27

0

0

0

26

3

0

3

0

17

17

6

6

1

0

0

0

0

0

0

1

0

1

0

0

0

217

14

10

10

0

0

0

0

4

4

0

0

14

2

2

12

0

0

12

0

0

0

0

9

0

0

0

0

9

9

0

0

0

0

0

0

1

1

1

32

29

29

3

3

0

0

0

0

147

0

0

53

0

0

0

37

0

16

15

0

0

10

5

9

9

0

70

0

22

0

8

27

0

0

0

0

3

5

3

2

0

0

0

0

0

0

0

0

0

0

0

0

0

0

0

0

0

0

0

0

0

0

0

0

0

0

0

0

0

0

0

0

0

0

0

374

316

23

23

0

0

12

0

12

11

11

0

0

0

0

0

0

0

0

0

0

0

0

0

0

0

0

0

0

293

277

277

277

16

16

16

0

0

0

0

58

43

43

14

14

0

24

24

5

1

4

15

15

15

15

0

0

0

0

0

0

0

0

6470

4

4

0

0

0

1

0

0

0

0

0

1

0

0

1

0

3

0

0

2

0

0

0

2

0

0

0

0

0

0

0

0

0

0

0

0

1

0

0

1

0

0

0

0

0

0

109

109

109

109

109

1254

56

56

0

0

0

0

56

56

0

0

0

0

0

0

0

0

0

0

0

0

143

29

29

29

22

22

22

1

1

1

0

0

0

0

0

0

91

91

91

320

11

11

11

6

6

6

30

30

30

6

6

6

231

231

231

36

36

36

1

1

1

1

734

48

0

0

0

43

0

43

5

0

5

0

158

158

2

13

143

201

19

1

18

148

0

148

34

15

19

327

0

0

0

0

327

327

1512

835

6

6

6

378

251

0

48

15

10

86

57

35

4

4

5

5

0

40

0

0

40

48

30

9

9

30

30

451

49

49

0

0

389

389

13

13

0

0

677

263

263

13

250

0

414

0

0

1

1

413

413

3563

0

0

0

0

0

0

0

0

0

0

0

0

0

0

0

0

1

1

1

1

18

18

1

1

0

0

2

2

0

1

1

0

0

3

3

0

0

0

0

0

3

0

2

1

3

3

5

5

0

0

0

0

12

12

12

1

0

11

0

0

0

0

9

1

1

0

1

0

0

0

0

6

6

0

0

0

6

2

1

1

0

0

0

1

1

0

0

27

27

8

8

0

19

1

3

1

0

0

1

0

13

0

0

246

119

23

23

94

94

2

2

15

2

2

13

13

1

1

0

1

0

0

0

0

0

0

0

111

5

5

13

13

93

93

7

4

4

4

0

0

0

3

3

3

0

0

0

14

14

3

0

0

3

11

2

9

0

0

0

0

0

0

0

0

0

0

0

15

15

15

15

0

0

0

36

36

1

1

35

1

3

6

1

19

0

5

321

21

7

7

0

0

2

2

0

0

0

0

0

0

0

0

0

0

12

0

12

0

0

0

300

10

3

0

7

5

5

268

0

1

0

0

0

0

100

9

2

5

19

10

5

0

47

2

0

0

10

42

0

15

1

0

0

17

3

14

0

0

22

22

1

1

0

0

1

0

0

0

1

0

0

6

0

1

0

0

5

0

7

0

1

3

3

0

0

3

3

4

4

2835

2321

1

1

11

11

282

0

282

0

0

0

0

0

1681

2

1160

42

350

127

3

3

0

0

343

343

35

0

0

5

5

4

4

9

9

0

0

0

0

5

5

12

12

0

0

0

257

236

236

0

0

21

21

192

4

4

0

0

8

8

6

6

0

0

0

0

0

0

0

0

0

0

0

9

9

4

4

6

6

141

1

27

113

9

9

1

1

0

0

4

4

0

0

0

30

24

24

6

6

28

28

28

28

6

0

20

2

45

44

44

44

0

0

6

0

0

0

6

0

0

0

0

0

0

0

0

0

0

0

0

0

0

0

0

0

0

0

38

38

0

0

0

0

0

0

0

1

1

1

1

1

592

592

592

592

592

592

32884

32884

32884

32877

32870

32870

0

0

0

0

7

6

1

7

7

0

0

0

0

0

7

0

0

0

49

49

49

35

35

0

1

34

0

0

14

14

0

14

0

0

0

0

0

0

0

0

0

0

45

0

0

0

0

0

24

24

21

21

21

3

0

0

3

3

21

21

0

0

0

7

7

7

14

14

14

13

13

13

13

13

4

9

0

0

542

216

7

7

7

7

209

103

84

77

7

1

1

0

0

18

18

106

39

39

1

1

0

0

66

66

109

15

15

15

10

5

0

0

3

3

3

3

0

0

0

91

91

0

0

36

0

17

19

13

0

13

27

3

15

9

15

0

0

15

15

0

0

0

0

15

15

15

15

0

0

0

0

58

58

58

0

0

58

6

0

0

0

37

6

0

9

0

0

0

0

0

144

144

21

21

21

117

117

117

6

6

6

0

0

0

0

1915

24

0

0

0

0

0

0

0

0

24

24

24

24

27

7

7

7

7

9

0

0

0

9

9

9

0

0

0

0

11

11

11

11

0

0

0

0

0

0

0

0

0

0

0

0

0

0

0

0

0

0

0

0

0

0

0

0

0

0

0

0

0

0

0

0

0

0

0

1864

1864

1294

1294

1294

38

38

38

0

0

0

84

67

67

17

17

8

8

8

257

0

0

242

0

242

0

0

0

0

0

0

15

15

53

53

53

130

130

130

31

31

31

0

0

0

31

31

31

0

0

0

0

0

0

0

0

0

0

0

0

0

0

0

0

0

0

0

0

133

90

13

13

9

9

4

0

4

0

0

0

0

77

51

51

51

18

18

18

0

0

0

0

0

0

8

8

8

0

0

0

0

0

0

0

0

43

35

35

12

12

23

0

23

8

8

8

0

8

39

39

39

16

16

0

16

0

23

7

0

7

16

2

0

0

0

0

0

14

0

0

0

0

0

0

0

0

3403

909

1

1

0

0

0

0

0

1

0

0

0

1

0

0

0

0

0

0

0

0

0

0

0

0

0

0

0

0

0

0

0

0

3

3

3

0

2

1

676

676

0

0

2

2

0

0

0

0

0

53

17

36

0

0

0

38

37

0

1

0

0

2

1

1

0

0

0

0

0

0

0

0

0

0

1

1

0

0

7

0

0

7

0

0

9

0

4

3

0

2

0

0

0

0

0

0

0

0

0

0

0

0

0

0

0

0

0

0

0

12

0

12

0

0

25

0

22

3

14

2

2

0

5

0

5

5

0

0

0

0

4

0

0

1

0

0

0

23

1

0

0

22

0

63

63

0

0

0

0

0

0

0

0

0

0

0

0

0

0

2

2

1

1

58

0

7

50

1

0

0

0

0

65

65

2

2

0

0

1

0

1

0

1

1

20

4

16

0

0

0

272

0

176

1

19

0

36

5

1

0

0

1

33

0

0

0

0

0

0

0

0

0

0

74

3

0

0

0

0

0

0

0

0

0

0

0

1

1

0

1

1

1

0

0

0

0

1

0

0

0

0

0

70

3

3

0

0

0

0

0

0

19

19

1

1

47

45

2

1

0

0

0

0

0

0

0

0

0

1

1

0

0

24

24

1

1

0

0

20

17

1

0

1

0

0

1

3

3

18

3

3

3

0

0

0

0

0

0

9

9

9

6

6

6

12

12

12

12

0

0

0

0

0

99

80

0

0

0

2

2

1

1

0

0

0

1

1

0

0

0

0

0

0

0

0

0

0

0

0

0

0

0

0

12

2

0

0

9

1

1

1

0

0

0

0

0

0

0

0

0

0

0

17

17

17

0

17

29

1

0

0

28

7

7

7

0

0

0

4

0

0

0

4

4

0

0

0

0

0

0

0

0

0

0

0

0

0

0

0

0

8

0

0

0

0

0

0

0

0

0

0

0

2

0

1

1

0

0

0

0

0

0

0

0

6

6

0

0

0

0

0

0

0

0

0

2

1

1

1

0

0

0

1

1

1

0

0

0

0

0

0

2392

0

0

0

0

700

202

0

0

0

0

1

1

22

0

1

0

21

0

0

0

0

85

85

21

21

73

73

0

0

498

0

0

0

0

0

0

0

498

0

7

0

83

7

13

33

0

0

18

277

2

23

18

0

7

10

0

0

0

0

9

9

9

9

0

0

0

0

0

166

64

0

0

0

0

62

62

1

1

0

0

0

0

0

0

0

0

0

0

0

0

1

1

102

3

3

25

25

3

1

2

0

0

0

19

19

0

0

0

0

0

0

0

13

0

13

28

28

10

10

0

0

1

1

0

0

0

0

0

0

0

0

0

0

24

24

5

5

1

1

18

4

14

0

0

0

0

0

0

0

0

0

0

298

298

9

9

8

8

241

21

161

0

0

1

0

9

0

1

0

0

0

6

0

25

0

0

1

14

2

0

0

0

0

0

0

0

0

12

12

28

0

23

5

5

1

1

1

0

0

0

4

4

4

0

0

0

559

559

559

558

1

618

6

6

5

1

31

15

0

0

15

0

0

1

1

15

15

160

155

0

32

0

0

66

0

40

2

0

0

0

15

0

5

5

0

1

1

0

1

419

0

0

2

2

7

6

0

1

2

0

1

1

230

2

86

123

0

0

19

18

18

21

20

0

0

1

0

0

5

5

106

0

0

106

5

1

4

14

14

9

2

7

1

1

1

0

0

0

0

0

0

0

6

6

6

6

0

0

0

0

0

7

7

7

7

0

0

0

0

0

0

0

0

0

8

8

8

8

8

0

0

0

0

0

67

0

0

0

0

0

0

67

0

0

0

0

0

0

0

0

0

0

0

0

0

0

0

0

0

0

0

0

0

0

0

67

62

62

0

0

0

5

5

0

0

0

0

0

0

0

0

0

27

27

27

27

27

129

18

11

11

11

11

0

0

0

0

0

7

7

7

7

0

0

0

0

0

0

0

0

0

0

13

0

0

0

0

13

5

5

5

8

8

8

0

0

0

0

29

20

20

20

20

1

1

0

0

0

1

1

0

0

0

6

6

6

6

0

0

0

0

0

0

0

0

0

0

0

2

0

0

0

0

0

0

2

2

2

69

69

69

69

69

27

27

27

27

27

1

26

223

223

223

223

223

113

110

0

0

0

0

0

0

6

6

6

0

0

0

0

0

0

0

0

0

6

6

6

0

0

0

0

0

0

0

0

0

0

0

0

0

12

4

4

4

0

0

0

4

4

4

7

7

7

7

7

7

0

0

1

1

1

1

1

1

0

0

0

0

0

0
